# The Thames and the Sewage

**Published:** 1896-11-14

**Authors:** 


### THE THAMES AND THE SEWAGE.

### A TRIP ON THE "BEATRICE."

The "Beatrice" is not an ocean-going steamer, nor is she a pleasure yacht; nevertheless, she is a tight little boat, well fitted for her work, capable of steaming at fifteen knots an hour, and able to twist and turn and wriggle her way with great facility through the crowded traffic of the Thames. The "Beatrice" is, in fact, the steam launch belonging to the County Council, used by those officials and members of committees who have to inspect the various works belonging to the Council, situated on one bank or the other of the mighty waterway.

Years ago the state of the river was a constant topic of conversation and a perennial source of grumbling. Metaphorically, the river was in everyone's mouth; literally, it was in everyone's nostrils. But we soon forget; and probably few people now ever think of the care and attention bestowed upon the state of the river as it flows through our great city. The "Beatrice" has a little cabin, placed forwards, which, on the occasion of our visit, was fitted up as a temporary laboratory.

We were going down the river to see what was being done with the London sewage, and to see for ourselves the results which are being obtained in some experiments which have been carried on for several years past under the direction of Mr. Dibdin, chemist to the County Council, with the object of dealing more satisfactorily and more economically with the sewage than is possible by present methods.

We had thought that an hour's run to Crossness would give ample time for the discussion of many things, but we were quickly undeceived. "Well," said Mr. Dibdin, "you must remember that we are pretty busy." And so it turned out to be. As we steamed quickly down the river, bottles of river water, sucked up from the depths, were constantly being brought down for examinations, and Mr. Dibdin's two assistants were pretty continuously occupied with their dishes and burettes making analyses and estimations, all of which were recorded as we went on, the place, the time, the tide, and the condition of the water being duly noted.

Perhaps it may be news to some to hear that even as the Thames runs through London large tributary streams enter the main river. They are all underground, but there they are. Running through the gravel on which all the older part of London stands is a slow-flowing river, perhaps as great as the Thames itself, and as the river level descends, the water from the gravels, flowing along channels excavated during long ages in the surface of the underlying clay, enters the main stream at various points. Thus, opposite the old Fleet, the river suddenly becomes much cleaner, being diluted by pure water flowing from the water-bearing strata which used to feed that ancient stream. On arriving at Crossness we saw the great pumping works, by the ceaseless action of which the sewage from the whole of the south of London is raised about eighteen feet, so as to flow into the Thames at the proper part of the tide. The sewage as it arrives is first mixed with lime water, the proper making of which is no inconsiderable affair. It is then treated with a small dose of iron sulphate, after which it is turned into the tanks—dark, undergound chambers—in which the mud which it contains is gradually deposited, leaving a comparatively clear effluent to flow into the river.

Immense, however, as is the improvement in the character of the effluent produced by the treatment it undergoes, it is by no means pure water; and moreover, with the constant growth of London, the tanks at Crossness and those at Barking, where the sewage from the northern part of London is discharged, vast as they are, by no means indicate finality in the arrangements which will be necessary for dealing with this great sociological problem—the disposal of the sewage of the metropolis. Hence the great financial importance of Mr. Dibdin's efforts to discover a better way.

Crossing over to Barking we found a repetition of the same arrangement as we had seen at Crossness, although carried out in a somewhat less perfect manner. The chemical and other laboratories also are of a makeshift character at Barking compared with those at Crossness. All this, however, is going to be altered. At Barking was the real object of our pilgrimage—the "experimental acre," on which for the last three years the foul ingredients of the sewage has day by day been eaten up by microbes, and the effluent from the London drains has, as the result of a carefully planned biological process, been converted into a clear brook in which fish can live, and up which they not infrequently find their way.

It is now five years since the Main Drainage Committee ordered a series of experiments to be made as to the best methods of filtering the sewage effluent obtained from the northern outfall precipitation works. After various preliminary experiments, a filter was constructed covering exactly an acre, the filtering medium being a layer of coke breeze three feet thick, covered with three inches of gravel, and on to it the effluent from the tanks was poured. The surface was at first kept constantly submerged, the object being to ascertain the rate at which it was possible to pass the effluent through. The result, however, was that the whole filter rapidly became clogged, the quantity which could be passed through rapidly fell, until at the end of twelve weeks the filter was quite useless, being putrid throughout. "Here was a disastrous ending!" some might say. But, in truth, it was just the opposite, for it was but proof, if any were needed, that a filter which only acted by mechanically separating foul particles must itself become foul. Clearly something more than mere mechanical straining was wanted if the filter was to maintain its activity.

After this the filter was left alone, in order that the impurities with which it had become clogged might rot away. For fully three months a putrid odour was observable whenever its surface was disturbed, but after a time this disappeared, and the coke breeze became quite sweet again. Then the filter was started afresh, and kept practically constantly at work for nearly a year without showing any signs of deterioration, since which it has been employed even more actively—in fact, it improved as it went on. But this time it was worked in a different way. Instead of being swamped in sewage the filter was emptied twice a day, air being drawn in as the fluid flowed out, and the whole of the interior of the filter being thus aërated afresh each time. Under this treatment its efficacy increased until it became capable of removing about 80 per cent. of the oxidisable material contained in the effluent; in fact, the latter became so clean that fish could be kept alive in it for many weeks, and minnows and sticklebacks came up the ditch to the very mouth of the outlet.

(*To be continued.*)

